# Keeping a watchful eye on the food giants and cleansing the temple of nutritional medicine and epidemiology

**DOI:** 10.1590/1516-3180.2017.136118131217

**Published:** 2018-03-05

**Authors:** Paulo Andrade Lotufo

**Affiliations:** I MD, DrPH. Full Professor, Department of Internal Medicine, Faculdade de Medicina da Universidade de São Paulo (FMUSP), São Paulo (SP), Brazil.

One of the most-loved enemies of medical editors, healthcare policy planners, scholars and scientists in the fields of medicine and public health is the companies that are known as “big pharma.” Indeed, our attitude is correct, because these companies keep on pushing products that lead to addiction to opioids and fentanyl (not unlike the addiction to heroin that is perpetrated by “traditional” drug dealers)^1^ and indulge in disease-mongering such as highlighting testosterone insufficiency in males.^2^^-^^3^ Two former editors of the *New England Journal of Medicine*, Marcia Angell and Jerome Kassirer, described in detail how “big pharma” works. Their reports did not receive any credible rebuttal from the pharmaceutical companies.^4^^-^^5^

However, over the last two decades, the scientific community has been curbing the bad behavior of “big pharma.” Governments have demanded that medical associations should implement compliance policies regarding publicization and commercialization of pharmaceutical products during congresses and events. For talks and papers, disclosure of conflicts of interest by lecturers and authors has become the reality, and there is no chance of turning the clock back. The most important stance certainly came from the International Committee of Medical Journal Editors, when it determined that all randomized clinical trials should be preregistered on public websites before the beginning of participant enrolment, such that this would be a *sine qua non* condition for further publication in any journal.^6^

While “big pharma” loves to push its products, most of these are, in reality, at breakthrough point, as has been seen in relation to statins. On the other hand, the community of editors, public health planners, scholars and scientists in the fields of medicine and public health has been negligent in relation to the actions of the food industry. The community has acted as well-wishers in relation to “alternative” diets. This attitude is erroneous and constitutes a typical double standard.

There is no reason why a critical attitude should not be adopted in relation to the food industry, alternative diets and published papers addressing diets that apply inadequate tools or meta-analyses. In contrast to current levels of understanding of the workings of big pharma, medical journals are less familiar with descriptions of how “big food” works.

The lay press has sometimes adopted a more critical approach than have scientific journals. One example can be seen in a book by Michael Moss called “Salt, sugar, fat: how the food giant hooked us,” which forms a summary of a series of articles by this author that were published in the New York Times.^7^ Recently, this newspaper published an excellent four-page description of how major companies like Nestlé operate in Brazil, with sales of high-caloric foods to poor people.^8^

Separately, but with similar bias, physicians, dietitians and physical educators have created a very profitable activity through selling books and cookbooks and giving very well rewarded lectures that mostly address foolish theories relating to diet.^9^ One of the worst examples of this comes from an overrated Brazilian physician who demonizes statins and at the same time uses this to sell his fad diet books. A review of 206 industry-funded studies found a a sevenfold greater chance of a favorable conclusion than of an unfavorable conclusion, in comparing those fully funded by the food industry with those without such funding, respectively.^10^ Furthermore, the same comparison showed a “bias factor” of 3.6 for funded studies!^11^

However, within the traditional scientific field of nutritional epidemiology, there is a terrible mess. Recently, a cohort study with dubious results regarding connections between fat and heart disease confidently claimed that the accumulated conclusions from decades of knowledge acquired through large observational studies should be dismissed. The authors’ conclusion was very pretentious, stating that “global dietary guidelines should be reconsidered in the light of these findings.”^12^

Meta-analyses have been useful for assessing issues relating to treatments, but they are not an appropriate tool for observational studies relating to diets used in different times and places.^13^ More than eight decades of studies showing that consumption of foods containing animal fat was associated with atherosclerosis and cardiovascular events were “invalidated” through a meta-analysis claiming that “butter is back” (in fairness, the authors did not state this,^14^^,^^15^ but it was consequently expressed thus on the cover of Time magazine).^16^ Barnard et al.^13^ concluded: “the food industry is well aware of the power of science-driven headlines and has invested in meta-analyses. In the process, nutritional science may be adversely affected.”

Interestingly, contestation of knowledge is a one-way street. In contrast to some innovative hypotheses such as NOVA,^17^ a proposal launched by Brazilian scientists was contested in a bitter editorial from the American Journal of Clinical Nutrition, in which the first author disclosed that he “serves on scientific committees for Nestlé and Cereal Partners Worldwide.”^18^ The journal did not accept the rebuttal from NOVA’s proponents, who needed to publish it elsewhere.^19^

In conclusion, we need to continue to keep a watchful eye on big pharma, while, at the same time, doing this with the same vigor in relation to the “food giants” and “cleansing the temple” of medicine and epidemiology relating to diet and nutrition.

## References

[1] Alexander B (2017). When a Company is Making Money from the Opioid Crisis. The Atlantic.

[2] Payer L (1992). Disease-Mongers: How Doctors, Drug Companies, and Insurers are Making You Feel Sick.

[3] Vitry AI, Mintzes B (2012). Disease mongering and low testosterone in men: the tale of two regulatory failures. Med J Aust.

[4] Angell M (2004). The Truth about the Drug Companies: How they deceive us and what to do about it.

[5] Kassirer JP (2005). On the Take: How Medicine's Complicity with Big Business Can Endanger Your Health.

[6] De Angelis C, Drazen JM, Frizelle FA (2004). Clinical Trial Registration: A Statement from the International Committee of Medical Journal Editors. Ann Intern Med.

[7] Moss M (2014). Salt, sugar, fat: how the food giant hooked us.

[8] Jacobs A, Richtell M (2017). How Big Business Got Brazil Hooked on Junk Food. The New York Times.

[9] Ioannidis JPA, Trepanowski JF (2017). Disclosures in Nutrition Research. Why is it Different?. JAMA.

[10] Lesser LI, Ebbeling CB, Goozner M, Wypij D, Ludwig DS (2007). Relationship between funding source and conclusion among nutrition-related scientific articles. PLoS Med.

[11] Bekelman JE, Li Y, Gross CP (2003). cope and impact of financial conflicts of interest in biomedical research: a systematic review. JAMA.

[12] Dehghan M, Mente A, Zhang X (2017). Associations of fats and carbohydrate intake with cardiovascular disease and mortality in 18 countries from five continents (PURE): a prospective cohort study. Lancet.

[13] Barnard ND, Willett WC, Ding EL (2017). The Misuse of Meta-analysis in Nutrition Research. JAMA.

[14] Chowdhury R, Warnakula S, Kunutsor S (2014). Association of dietary, circulating, and supplement fatty acids with coronary risk: a systematic review and meta-analysis. Ann Intern Med.

[15] Pimpin L, Wu JH, Haskelberg H, Del Gobbo L, Mozaffarian D (2016). Is Butter Back? A Systematic Review and Meta-Analysis of Butter Consumption and Risk of Cardiovascular Disease, Diabetes, and Total Mortality. PLoS One.

[16] Dehghan M, Mente A, Zhang X (2017). Associations of fats and carbohydrate intake with cardiovascular disease and mortality in 18 countries from five continents (PURE): a prospective cohort study. Prospective Urban Rural Epidemiology (PURE) study investigators. Lancet.

[17] Monteiro CA, Cannon G, Moubarac JC (2018). The UN Decade of Nutrition, the NOVA food classification and the trouble with ultra-processing. Public Health Nutr.

[18] Gibney MJ, Forde CG, Mullally D, Gibney ER (2017). Ultra-processed foods in human health: a critical appraisal. Am J Clin Nutr.

[19] Monteiro CA, Cannon G, Moubarac JC (2017). Ultra-processing. An odd 'appraisal'. Public Health Nutr.

